# IL-1 in Abdominal Aortic Aneurysms

**DOI:** 10.33696/immunology.5.163

**Published:** 2023

**Authors:** Jessica Millar, Elias Nasser, Gorav Ailawadi, Morgan Salmon

**Affiliations:** 1 Department of Surgery, Michigan Medicine, University of Michigan, Ann Arbor, Michigan, USA; 2 Department of Cardiac Surgery, Michigan Medicine, University of Michigan, Ann Arbor, Michigan, USA; 3 Frankel Cardiovascular Center, University of Michigan School of Medicine, Ann Arbor, Michigan, USA

**Keywords:** AAA, IL-1α, IL-1β, Anakinra

## Abstract

Abdominal Aortic Aneurysms (AAA) remain a clinically devastating disease with no effective medical treatment therapy. AAAs are characterized by immune cell infiltration, smooth muscle cell apoptosis, and extracellular matrix degradation. Interleukin-1 (IL-1) has been shown to play role in AAA associated inflammation through immune cell recruitment and activation, endothelial dysfunction, production of reactive oxygen species (ROS), and regulation of transcription factors of additional inflammatory mediators. In this review, we will discuss the principles of IL-1 signaling, its role in AAA specific inflammation, and regulators of IL-1 signaling. Additionally, we will discuss the influence of genetic and pharmacological inhibitors of IL-1 on experimental AAAs. Evidence suggests that IL-1 may prove to be a potential therapeutic target in the management of AAA disease.

## Introduction

Aortic Aneurysms are defined as localized, full-thickness dilation of the aorta related to regional weaking of the wall structure [1,2]. They can be classified into three sub-types based on location along the aorta: ascending aortic aneurysm (AA), descending thoracic aortic aneurysm (dTAA), or abdominal aortic aneurysm (AAA) [3]. Thoracic aortic aneurysms (AA and dTAA) are more likely to develop due to genetic syndromes (such as Marfans, Loey Dietz, or Ehlers-Danlos) or genetic predisposition (Bicuspid Aortic Valve or familial Thoracic Aortic Aneurysm and Dissection) and are more prone to dissection [2,4,5]. AAAs, however, are more likely to develop due to local hemodynamic patterns and associated wall stress [6]. Other risk factors, such as history of smoking, biological sex, age, and family history, have also been implicated in the development of AAAs [2,4]. Smoking in particular is associated with larger aneurysm size at diagnosis as well as a higher risk of aneurysm progression [7–10].

The natural history of AAAs is that of slow progression of size and ultimate rupture [5,11,12]. In contrast to thoracic aortic aneurysms, AAAs are more likely to have spontaneous rupture which carries an 80-90% mortality rate and accounts for approximately 13,000-15,000 deaths per year in the United States [2,4]. The risk of AAA rupture is directly related to the maximal aortic dilation with an estimated annual risk of rupture of 1% for AAAs greater than 5 cm in diameter and up to 30% for AAAs that exceed 8 cm in diameter [1,2,13]. Current guidelines recommend repair, either surgically or endovascularly, for AAAs with an aortic diameter of 5.5 cm for men and 5.0 cm in women in order to balance the risk of intervention with the risk of rupture [1,2,6,13]. However, AAAs may rupture at sizes smaller from currently unknown mechanisms [2,4]. Screening recommendations and improvements in imaging technology have helped increase detection of early-stage AAAs. Yet, in the absence of effective medical therapy, patients diagnosed with small AAAs must wait to undergo intervention until the aforementioned size criteria is met and are subjected to continuous monitoring and surveillance [14].

Despite an ever-growing body of research, the mechanisms that contribute to AAA progression and rupture remain poorly understood and, as such, there is currently no standard for medical management of small AAAs or for AAAs in patients unable to undergo intervention [1,2,15]. Current strategies include treatment of hypertension, optimal lipid control, and smoking cessation. However, these are generally seen as strategies to improve overall cardiovascular health and are not specifically targeted at AAA disease [15]. Elective and emergent AAA interventions account for more than 15,000 surgical procedures annually, thereby placing a large burden on our current health care system [2,4,16–19]. Medical therapy could help stabilize small diameter aneurysms and prevent or reduce the need for surgical repair. Additionally, it could serve as definitive therapy in patients considered high risk for surgical repair [1].

The main pathological driving factors of aortic aneurysm formation include infiltration of the vessel wall by inflammatory cells (lymphocytes, macrophages), destruction of elastin and collagen resulting from metalloproteinases, loss of smooth muscle cells, increased activation of pro-inflammatory cytokines, augmented oxidative tissue damage, and neovascularization [2,20,21]. From these, common themes appear including increased inflammation and altered extracellular matrix metabolism [11]. Interleukin-1 (IL-1) has been demonstrated to play a key role in vascular inflammation, including AAAs. In this review, we will discuss the role of IL-1 signaling in AAA disease and how inhibition, through genetic and pharmacological means, has demonstrated IL-1 to be a promising pathway for the medical treatment of AAA disease.

### Interleukin-1α and 1β

IL-1 is an inflammatory cytokine with diverse physiologic and pathologic effects and plays an important role in both health and disease [22]. IL-1 is known as a master regulator of inflammation, controlling a variety of innate immune processes, such as mediating fever response [23,24]. IL-1 is expressed in a wide range of tissues and cells including macrophages in the thymus, bone marrow, lung, and liver as well as neutrophils, keratinocytes, endothelial cells, smooth muscle cells, and fibroblasts [22]. Today, there are 11 total recognized members of the IL-1 family each with similar or distinct biological effects [22].

IL-1 was first isolated from human monocytes and neutrophils and described as “acidic and neutral human pyrogens” collectively called “Interleukin 1”. It was ten years before these proteins were identified as being distinct at the amino acid level and were subsequently renamed IL-1α and IL-1β [25]. IL-1α and IL-1β are encoded by different genes that bind to the same receptor (IL-1R) to activate a proinflammatory pathway [26]. While they share only 24% of their amino acid sequence, they are indistinguishable in terms of their biologic function [22,27–29]. However, the factors that control their functional maturation and bioavailability are highly dissimilar and differences between their cellular source, maturation requirements, and release impact their role in inflammation [25,26].

IL-1α is constitutively expressed by many cell types in healthy tissues at a steady state. For example, barrier cells, such as endothelial and epithelial cells, express substantial amounts of IL-1α during steady state [28,30,31]. However, its expression can be increased in response to growth factors and pro-inflammatory/stress-associated stimuli such as oxidative stress, lipid overload, hormonal stimulation, and exposure to cytokines (including IL-1β and IL-1α itself) [25]. IL-1α is a primarily membrane anchored protein, however, it functions as both a secreted and as a membrane-bound cytokine and signals through autocrine or juxtracrine mechanisms [29,32].

Conversely, IL-1β is not constitutively expresses and is absent in cells at homeostasis. IL-1β mRNA is expressed upon activation only in cells of hematopoietic origin and requires an additional signal including microbial products or other cytokines including IL-18, TNFα , IL-1α or IL-1β itself [26,30]. The major sources of IL-1β secretion include macrophages, monocytes, dendritic cells, B lymphocytes, neutrophils, and natural killer cells [24,33]. In contrast to IL-1α, IL-1β is a secreted protein and exerts its effects in a largely paracrine or systemic mechanism [29].

Both IL-1α and IL-1β are produced in pro-forms (pro-IL1α and pro-IL1β) and are later cleaved through various activation processes. Whereas only the cleaved form of IL-1β is functional, both pro-IL1α and the cleaved form of IL-1α are biologically active and activate the IL-1 receptor-1 (IL-1R1) with identical biological activities [34].

### IL-1 Signaling

IL-1 induces the mRNA expression of hundreds of genes in multiple different cell types including macrophages, endothelial cells, and fibroblasts [29]. Additionally, IL-1 also stimulates its own gene expression in a positive feedback loop that amplifies the IL-1 response in an autocrine and paracrine manner [22,29]. This loop of sustained, self-perpetuating inflammation results in extensive tissue damage that occurs until IL-1 signaling is either exhausted or suppressed [25]. Although the regulation and effects of IL-1β have been extensively studied, most aspects of IL-1α biogenesis and function in the inflammatory process remain largely unknown [30,31,33,35]. As this review is primarily focused on IL-1β as it relates to AAA disease, we will focus on the specifics on IL-1β signaling.

IL-1β signaling begins with the synthesis of the biological inactive IL-1β precursor, pro-IL-1, by nuclear factor kappa B (NF-κB) binding to transcribe the IL-1β gene [36,37]. Pro-IL-1β is then processed into mature, biologically active IL-1β by caspase-1 activated by the inflammasome [38]. However, IL-1β can also be processed by other serine proteases such as elastase, chymases, granzyme A, cathepsin G, and proteinase-3 [39].

IL-1β acts primarily on IL-1R1 expressed by T-lymphocytes, fibroblasts, epithelial cells, and endothelial cells. After IL-1β binding, IL-1R1 forms a heterodimer with IL-1R3 and is accompanied by the IL-1 receptor accessory protein (IL-1RAcP) [40]. The adaptor IL-1 receptor associated kinase (IRAK) and myeloid differentiation primary repones protein 88 (MyD88) are recruited to this complex to form a stable IL-1-induced first signaling molecule [22,29,41]. This complex will go on to activate NF-κB which will lead to the expression of IL-1 responsive genes including IL-6, IL-8, monocyte chemoattractant protein 1 (MCP-1) and cyclooxygenase 2 (COX2) [29].

A second IL-1 receptor, IL-1 receptor-2 (IL-1R2), exists largely as a decoy receptor and is thought to reduce the biological response to IL-1β as it does not contain a signaling-competent cystolic portion [22,29]. Expression levels of IL-1R1 and IL-1R2 are different among different cell types with IL-1R2 being primarily expressed on neutrophils, B-lymphocytes, and bone marrow cells. As a result, these cells often require a much high concentration of IL-1β for activation. Conversely, endothelial cells predominately express IL-1R1 and require low concentrations of IL-1β for activation [42,43]. To further highlight their signaling differences, IL-1α has a higher affinity for IL-1R1 while IL-1β has been demonstrated to have a higher affinity for the decoy receptor IL-1R2 [24,33].

### IL-1 Signaling in AAA Disease

IL-1 signaling has long been proposed as a key inflammatory mechanism for AAA formation and progression. Previous murine models of AAA have demonstrated increased IL-1β mRNA and protein levels [27]. Likewise, in human AAAs, IL-1β gene and protein expression has been demonstrated to be increased 10-fold and 4-fold, respectively [27].

IL-1 signaling in AAA disease was once thought to be related to atherosclerosis. IL-1 is widely expressed in human and experimental atherosclerotic lesions with IL-1β playing a major role in the progression and rupture of atherosclerotic plaques; however, recent studies suggest IL-1 could stabilize advanced plaque formation [44–46]. One of the earliest steps of atherosclerosis is the recruitment of leukocytes by endothelial cells through the expression of adhesion molecules (such as ICAM-1 and VCAM-1) induced by IL-1β [23]. However, while IL-1 has been demonstrated to play an important role in the development of atherosclerosis, many questioned whether the same inflammatory pathways proven essential for atherosclerosis are also key in AAA [47].

Smoking (nicotine-exposure) is one the strongest associated risk factors for AAA progression and the main indicator for AAA screening [47]. Like the processes seen in atherosclerosis, nicotine upregulates ICAM-1 and VCAM-1, thereby recruiting leukocytes and activating the production of IL-1β by macrophages. In both processes, recruitment of myeloid cells to the aortic wall plays a critical role and highlights IL-1’s role in AAA disease through innate immune activation.

### Leukocyte signaling

**Figure 1.**
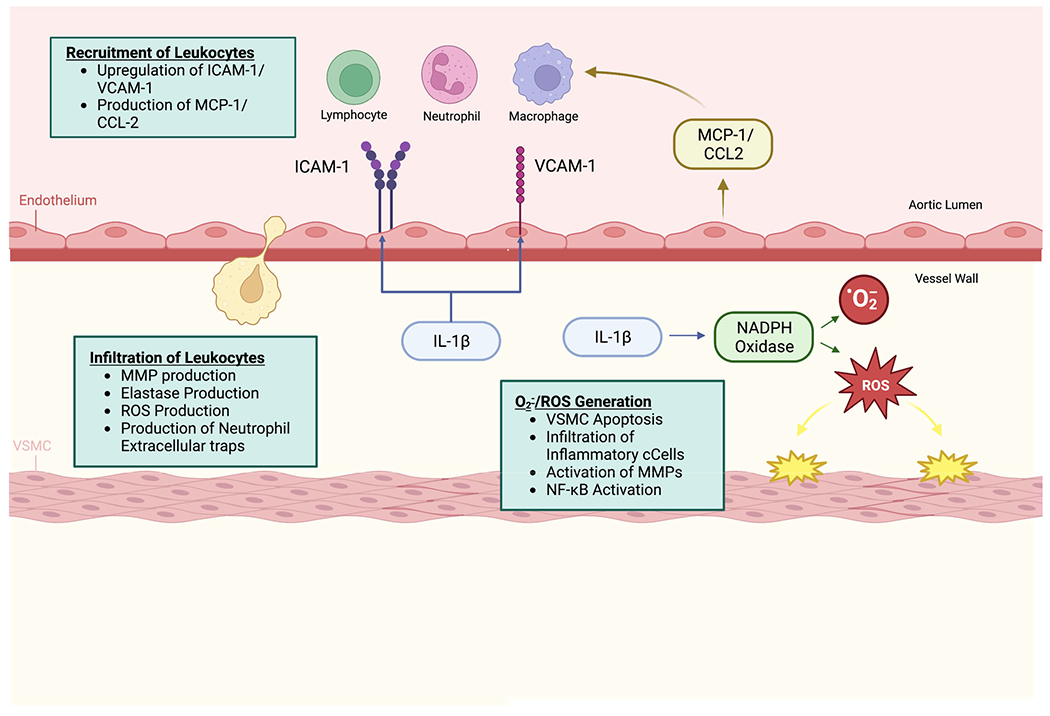
Model of Extracellular Functions of IL-1 in Aortic Aneurysm Formation.

AAA disease is often accompanied by a robust inflammatory response in the wall of the abdominal aorta with multiple different subsets of immune cells (such as monocytes, macrophages, neutrophils, dendritic cells, natural killer cells, and T-cells) accumulated within the tunica media and adventitia (Figure 1) [47]. Both IL-1α and IL-1β are believed to be key mediators in this response. IL-1α’s function depends on its sub-cellular location, regulating normal gene expression when expressed within the cytosol during homeostasis [48]. However, in the presence of cell death, passive leakage of cytosolic IL-1α may occur. This abundance of released IL-1α results in robust inflammation in an IL-1R1 dependent manner leading to its designation as a key “alarmin” in the cell that alerts the host to injury or damage [30,31,35,49]. IL-1 is known to upregulate adhesion molecules on endothelial cells, which in turn recruits immune cells in and around the aortic wall. The consequent inflammatory response aggravates AAA formation [21]. As a genetic-proof of principle, mice with IL-1 or IL-1R1 deletion have demonstrated less macrophage staining within the wall of the aorta after aortic aneurysm induction [26]. IL-1β also leads to the formation of neutrophil extracellular traps and neutrophil elastase release resulting in vast degradation of the extracellular matrix within the aortic wall [50].

Chronic inflammatory cell infiltration within the damaged aortic wall is largely dominated by pro-inflammatory CD4 T-cells and activated macrophages [6]. These cells can undergo phenotypic modulation based on their surrounding microenvironment to a largely pro-inflammatory phenotype, thereby influencing disease progression [6]. CD4 Th17 cells are stimulated by IL-1 and promote macrophage recruitment to the vascular wall. Deficiency in CD4 Th17 cell signaling has been demonstrated to reduce aortic macrophages in murine models [51,52]. Both of these myeloid cells contribute to AAA disease through matrix metalloproteinases (MMP) and elastase production, thus initiating destruction of the aortic wall resulting in aneurysm formation. In return, dying cells within the aortic wall release damage-associated molecular patterns (DAMPs) which are sensed by the accumulated myeloid cells, resulting in their continued activation and production of chemokine and inflammatory cytokines. Additionally, these infiltrating immune cells release reactive oxygen species (ROS) and induce expression of cellular adhesion molecules which lead to further recruitment of immune cells, induction of vascular smooth muscles cell apoptosis, and tissue injury [53].

### Macrophages and IL-1 signaling

Macrophages have been implicated as a key component of the inflammatory process in AAA disease through their production and activation of inflammatory cytokines as well as serving as a major source of MMP production [54,55]. Cell damage caused by endogenous stimuli results in a “sterile” inflammatory process and release of DAMPs. These signals ultimately lead to the activation of innate immune cells, primarily macrophages. Activated aortic wall macrophages subsequently initiate inflammasome activation and IL-1 production. In turn, IL-1β is a potent macrophage-inducer and results in their continued activation and signaling [54,55]. Additionally, IL-1 signaling has been linked to increase macrophage infiltration in AAAs [27]. This increased inflammatory cell accumulation inevitably leads to continued pro-inflammatory cytokine and chemokine release and further activation of MMPs and caspase production resulting in aortic wall degradation, loss of smooth muscle cells, and untimely further aneurysmal dilation [47].

IL-1β serves as a “risk” signal for smooth muscle cells within the aortic wall and has been shown to co-localize with aortic smooth muscle cells early in AAA formation [27]. IL-1β induces recruitment of innate immune cells by activation of monocyte chemoattractant protein-1 (MCP-1/CCL2) [16]. MCP-1/CCL2 is a potent chemoattractant that results in significant migration of monocytes/macrophages to inflammatory sites. Macrophages initiated by MCP-1 are more cytotoxic and have been demonstrated to induce higher levels of SMC apoptosis [56].

IL-1 signaling also leads to the activation of the c-Jun NH2-terminal protein kinase (JNK) pathway through toll-like receptors (TLRs) expressed on immune cells in AAA. This pathway has been demonstrated to promote AAA development by inducing pro-inflammatory chemokine release [41,50]. Inhibition of the JNK pathway has been shown to reduce MMP production and chemokine-mediated macrophage migration, thereby slowing the progression of AAA development in rats and humans [50].

### IL-1 Dependent Signaling

**Figure 2.**
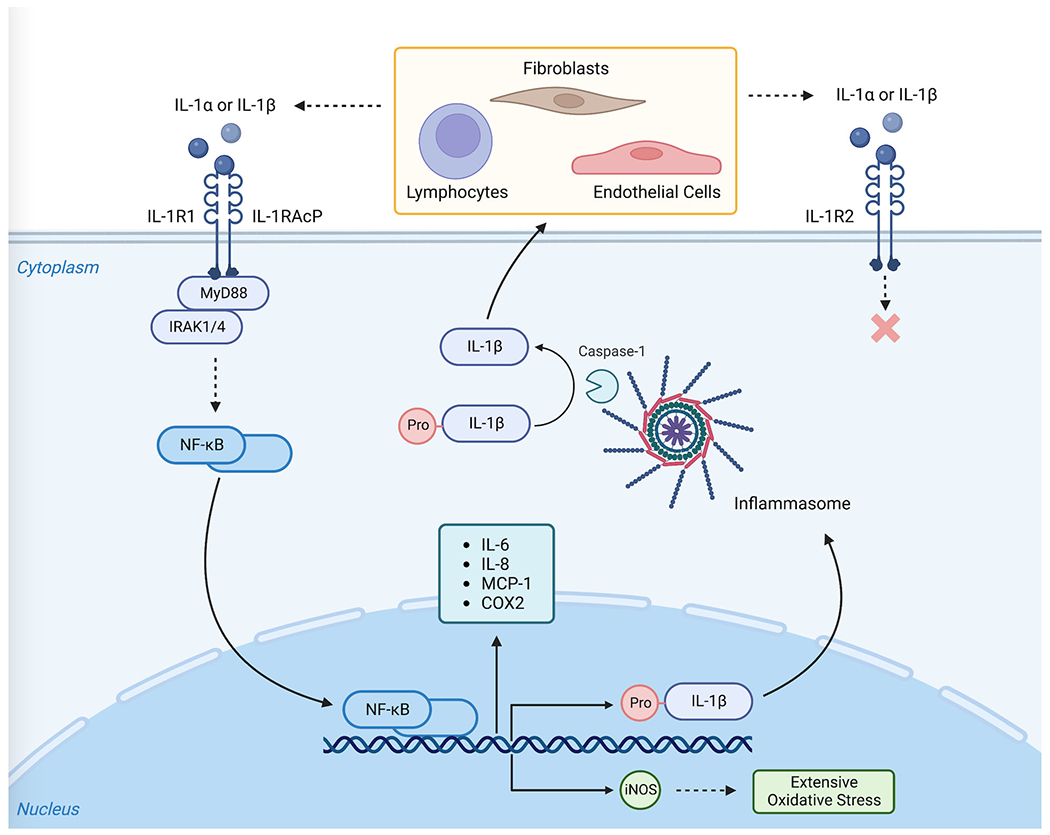
Model of Intracellular functions of IL-1 signaling in Aortic Aneurysm Formation.

In addition to innate immune activation, IL-1 signaling had been demonstrated to induce a number of cellular changes implicated in AAA disease (Figure 2). Endothelial dysfunction plays a large role in AAA formation and progression and is influenced by IL-1β signaling. Early endothelial dysfunction is due to IL-1R1 mediated activation of NADPH oxidase which enhances superoxide anion (O_2_^−^) production and excessive ROS generation [23,57,58]. Elevated and sustained levels of ROS induce vascular smooth muscle cell (VSMC) apoptosis resulting in depletion of cellular content of the medial layer within the aortic wall [50]. Additionally, ROS promote the infiltration of inflammatory cells, increase the secretion of pro-inflammatory cytokines, and can directly activate MMPs [57]. NADPH produced ROS also result in NF-κB- activation and inducible nitric oxide synthase axis (iNOS) activation [23,57,58].

iNOS can result in massive generation of nitric oxide (NO) resulting in extensive oxidative stress and inflammation. Thus, excessive NO generation can be an important factor in local destruction of the extracellular matrix through destruction of elastic fibers and cytotoxic effects on surrounding cells including marked apoptosis of VSMCs [58]. In addition to NADPH/ROS induced iNOS production, IL-1β can also directly activate the ERK 1/2 NF-κB- iNOS axis in human VSMCs [23]. Likewise, infiltrating inflammatory cells in AAA serve as another source of iNOS, mainly macrophages and T and B lymphocytes [58].

In the presence of such large-scale inflammation, the aortic wall undergoes significant weakening compounded by oxidative stress, VSMC apoptosis, and extra cellular matrix (ECM) remodeling. The presence of NADPH oxidases, the abundance of ROS, and the upregulation of iNOS induced by IL-1 signaling result in continued activation of ECM degrading enzymes and VSMC apoptosis [50].

IL-1β is a major activator of the transcription factor NF-κB which further amplifies the inflammatory reaction via transcription of several genes associated with both inflammatory and oxidative reactions within the aortic wall [57]. IL-1β NF-κB stimulation leads to the release of NF-κB from complexes with its inhibitory protein, IκBα, which allows NF-κB subunits to translocate to the nucleus to promote transcription of target genes [59]. NF-κB activation in SMCs sustains IL-1β production, thereby establishing an autocrine mechanism which further stimulates inflammatory cell infiltration and oxidative stress, thereby creating a vicious cycle [29,57]. Inhibition of the NF-κB pathway in endothelial cells has been shown to attenuate angiotensin II-induced AAA formation in murine models by reducing macrophage infiltration, oxidative stress, and aortic inflammation [50].

Through the aforementioned JNK and ERK pathways, IL-1β is known to increase the expression of MMP-1 and MMP-13. MMP-1 in particular was found to be significantly upregulated in aneurysmal aortic specimens comparted to healthy aortic tissues [50]. Independent of these pathways, IL-1β also increases the expression of MMP-8 [50]. Collectively, MMP activity is known to initiate ECM degradation and proteolysis within the aortic wall thereby contributing to aneurysmal degeneration [50,57].

### IL-1α Specific Signaling

Unlike IL-1β which has been extensively studied, little is known about the role of IL-Iα in AAA disease. One recent study demonstrated that IL-1α may help to attenuate AAA as IL-1α knockout AAA murine models were demonstrated to have larger AAA size compared to controls [48]. IL-1α knockout was also demonstrated to result in increased elastin breaks, increased levels of inflammatory macrophages and neutrophils, and increased MMPs [48]. The results of this study show that IL-1α and IL-1β may play separate, rather than overlapping roles, in AAA disease and that further studies specifically evaluating the role IL-1α in AAA disease are necessary [48].

### Regulators of IL-1 Signaling

While IL-1 has been implicated to play an integral role in AAA disease through mediation of inflammation, it is important to recognize that regulation of IL-1 signaling may also have a significant influence on AAA formation and progression. Inflammasomes are a multiprotein complex that are responsible for the cleavage of pro-caspace-1 into active caspase-1, which in turn convers the cytokine precursor pro- IL-1β into the potent proinflammatory mediator IL-1β [60]. The nucleotide-binding domain (NOD)-like receptor protein 3 (NLRP3) inflammasome is the main regulator of IL-1β. The NLRP3 inflammasome has been theorized to contribute to several human diseases, including cardiovascular disease [10,46].

Expression of the NLRP3 inflammasome is induced by multiple factors, most notably by tumor necrosis factor and IL-1β through the activation of NF-κB [60]. The NLRP3 inflammasome has been reported to be activated by a wide range of PAMPs and DAMPs including glucose, β-amyloid, and cholesterol crystals [22]. Chronic exposure to high levels of free fatty acids and glucose have been reported to induce NLRP3 inflammation resulting in increased apoptosis and impaired insulin secretion in pancreatic β-cells in type 2 diabetes through significant IL-1β production by infiltrating macrophages [22]. Similarly, previous studies have demonstrated enhanced expression of IL-1β in a high glucose milieu in human monocytes and macrophages, pancreatic islet cells, myocardium, and aortic endothelium thought to be due to increased NLRP3 activation [23]. However, some have theorized that the inflammasome detects disturbances in cellular homeostasis (such as K^+^ efflux, Ca^2+^ signaling, mitochondrial dysfunction, and lysosomal rupture) rather than directly recognizing common motifs present in these activators. As such, the NLRP3 inflammasome may become activated due to common cellular signals induced by its activators [60].

The main mechanism through which the NLRP3 inflammasome exerts its inflammatory effects is through the activation of caspase-1 which in turns cleaves pro- IL-1β and pro-IL-18 into their activated forms. Caspase-1 deficiency in ApoE (−/−) mice has been shown to reduce the diameter, incidence, and severity of AAA along with adventitial fibrosis and inflammatory responses [61,62]. However, some studies suggest that mature IL-1β may also be produced independent of caspase-1, especially in the context of local inflammation [63].

Other implicated regulators of IL-1 signaling include the microbial and viral components of the “microbiome”. Current studies are evaluating the microbiome as a distant regulator of cytokine induction and differentiation of cytokine producing cells. Small changes in the host microbiome have been associated with the development of various inflammatory disease such as colitis, obesity, and cardiovascular disease. It is possible that pathological activation of immune cells driven by bacterial products could promote AAA [47].

Perivascular adipose tissue which surrounds the aorta may also impact IL-1 signaling and aortic wall homeostasis. It has been suggested that inflammation in the perivascular adipose tissue has the ability to expand to the aortic wall and, as a result, contribute to AAA development [47]. This is the premise for adventitial elastase application models of AAA disease in murine models. Surrounding adipocytes in a pro-inflammatory environment become activated and produce pro-inflammatory cytokines, such as IL-1, which in turn facilitate immune cell activation [47].

TNF-α, another important protein in the regulation of both acute and chronic inflammatory responses, promotes NF-κB, IL-1β, IL-6, MMP-2, and MMP-9 levels in Angiotensin-II induced vascular smooth muscle cells [64].

### Other Signaling Effects of IL-1

Endothelial cells display remarkable heterogeneity in their response to exogenous stimuli. Some studies have suggested that vascular endothelial cells exposed to various environmental stimuli undergo dynamic phenotypic switching that results in endothelial cell dysfunction and, as a result, cause a variety of diseases [65]. Endothelial to mesenchymal transition (EndMT) is a complex biological process in which endothelial cells lose their endothelial characteristics, acquire mesenchymal phenotypes, and express mesenchymal cell markers. This leads to a loss of normal endothelial cell function in maintaining vascular homeostasis (such as permeability) and results in a pathological state including tissue fibrosis and atherosclerosis [65].

EndMT is induced by inflammation with both IL-1β and TGF-β being implicated as the main driving factors [59]. While there have been no studies directly examining the role of EndMT in AAA disease, *in vitro* studies have demonstrated that IL-1β stimulation leads to endothelial monolayer disruption and induces EndMT-like changes in endothelial cells upon long-term treatment [59]. Several studies have also implicated the NLRP3 inflammasome, the main regulator of mature IL-1β secretion, in the process of EndMT as well [65].

VSMCs are also able to respond to local environmental factors with tremendous plasticity and can change their phenotype to a proliferative/inflammatory state [66]. Similar to endothelial cells, IL-β modifies the expression of specific SMC genes relevant for ECM composition and cell adhesion, thereby altering the mechanical properties of the arterial wall which may contribute to AAA disease [67].

### Genetic and Pharmacologic Inhibition of IL-1 in AAA Disease

#### Genetic inhibition

Murine models have remained the best available strategy to study the molecular mechanisms for AAA disease [47]. Murine models of AAA with IL-1β or IL-1R1 knockout have demonstrated attenuated AAA formation with IL-1β knockout demonstrating the greatest protection [27]. These studies have demonstrated the role of IL-1 in progression of small established AAAs [27]. NLRP3 and IL-1β deficiency in ApoE (−/−) mice was also demonstrated to decrease the maximal diameter, severity, and incidence of AAA along with adventitial fibrosis and inflammatory responses [53,61]. Similar effects of IL-1β or IL-1R1 knockout have been observed in murine models of TAA and were demonstrated to have reduced accumulation of macrophages and neutrophils, fewer inflammatory cytokines, and lower MMP-9 levels [26,68].

#### Pharmacologic inhibition

Given its diverse and integral role in AAA disease and aortic wall inflammation, IL-1β inhibition has been proposed as a possible strategy for targeted medical therapy of AAA disease. Neutralization of IL-1 has been demonstrated to be safe in humans and has already been utilized and shown to have widespread benefit in autoinflammatory conditions such as gout, rheumatoid arthritis, and autoimmune pericarditis [22]. There currently exist multiple pharmacological agents which disrupt IL-1 signaling that are FDA approved [27].

Anakinra is a recombinant human intrinsic IL-1 receptor antagonist (IL1-Ra) and was the first biological drug of selective IL-1R1 antagonism to receive approval from the US FDA. It can prevent the activity of both IL-1α and IL-1β by blocking their binding to IL-1R1 [22]. Differences seen in IL-1α and IL-1β signaling in murine AAA models suggest that targeting of these molecules could produce different effects and that targeting their common receptor, IL-1R1, might be the preferred target for pharmacologic inhibition studies [48]. Murine models of AAA treated with escalating doses of the IL-1 receptor antagonist, Anakinra, demonstrated a dose-dependent decreased in maximal aortic dilation [27]. Similarly, murine models of AAA treated 3-7 days following AAA initiation demonstrated protection against AAA progression and attenuated AAA dilation [27].

Rilonacept is a soluble decoy receptor that binds both IL-1 and IL-1β with high affinity and prevents their activity with a long-term inhibitory effect. Similar to Anakinra, Rilonacept was approved by the FDA in 2008 [69]. Lastly, Canakinumab is a human monoclonal IgG1 antibody with affinity for IL-1β. It does not react with IL-1α or IL-1R1 and is a specific inhibitor of IL-1β. While not currently FDA approved, early clinical trials have demonstrated canakinumab to be safe and effective against several inflammatory diseases [70]. Additionally, clinical trials (NCT02007252) further evaluating the role of IL-1β inhibition on the expansion of small AAA in humans utilizing Canakinumab are currently underway [71]. Studies utilizing Canakinumab will help elucidated whether inhibition of IL-1β or its common receptor (IL-1R1) will yield the greatest protection in AAA formation and progression [26].

## Conclusion

In summary, there is an overwhelming and ever-growing body of evidence to suggest the diverse and integral role of IL-1 signaling in aortic wall inflammation and AAA disease. One area of continued study is that of regional specific signaling. Structural differences, differences in cell origin, regional diversity in microvascular endothelium, and immunologic makeup may result in differences in susceptibility for aneurysmal disease between the AAs, dTAAs, and AAAs. These may lead to differences in cellular responses to the same stimuli and regional variations in success for medical management. As such, studies should be conducted in these segments separately to evaluate these differences [57]. Additionally, further studies investigating the upstream regulators of IL-1 signaling (such as the NLRP3 inflammasome) may provide new insight into novel pharmacological targets for the treatment of AAA disease. As AAA remains a clinically relevant disease, there is an unmet need for effective medical management and IL-1 signaling may prove to be an effective pathway for targeted medical therapy.

## Non-standard Abbreviation and Acronyms

AAA: Abdominal Aortic Aneurysm
ACTA2: Smooth muscle α-actin
ECM: Extracellular Matrix
EndMT: Endothelial to Mesenchymal Transition
IL-6: Interleukin 6
IL-17: Interleukin 17
IL-23: Interleukin 23
IL-1α: Interleukin-1 alpha
IL-1β: Interleukin-1 beta
IL-1R1: Interleukin-1 Receptor 1
IFNγ: Interferon gamma
MCP1: Monocyte Chemoattractant Protein 1
MMP2: Matrix Metalloproteinase 2
MMP9: Matrix Metalloproteinase 9
MYH11: Smooth muscle cell myosin heavy chain
NF-B: Nuclear Factor Kappa Beta
SM22α: Transgelin
TNFα: Tumor Necrosis Factor alpha
VSMC: Vascular Smooth Muscle Cell
